# A service evaluation of the “Open Wide and Step Inside” school oral health programme

**DOI:** 10.1038/s41405-019-0013-0

**Published:** 2019-03-21

**Authors:** Robert Witton, Wendy Smith

**Affiliations:** 1 Faculty of Medicine and Dentistry, Peninsula Dental School, University of Plymouth, Plymouth, United Kingdom; 2 Peninsula Dental Social Enterprise, Plymouth, United Kingdom; 3 Well Connected, Plymouth, United Kingdom

## Abstract

**Introduction:**

To present a service evaluation of the “Open Wide and Step Inside” oral health programme delivered in Plymouth, UK.

**Aim:**

To develop an oral health programme supporting Key Stage One of the National Curriculum for delivery to children aged 4–6 years in targeted schools.

**Methods:**

The programme was designed and developed in partnership with stakeholders from a range of sectors using a community engagement approach. The programme has been delivered in schools in targeted areas of the city since 2014/15. Outcome (participation of schools and children) and process evaluations were carried out using a range of methods to collect feedback from children, teachers and parents.

**Results:**

School adoption and engagement in the programme has been high exceeding the targets set for implementation. So far over 4000 children have participated in the programme and schools have welcomed support in delivering oral health as part of the national curriculum.

**Conclusion:**

A community engagement approach can be highly valuable in developing oral health programmes that meet community needs through collaborative design and a participatory approach.

## Introduction

Tooth decay is largely preventable yet it remains a serious public health problem in children. In 2017 almost a quarter of five-year-olds had experienced tooth decay, having on average 3 or 4 teeth affected.^1^ Dental decay is the most common reason for hospital admission for five to nine-year old children in England.^2^ Poor oral health in children can lead to unnecessary pain and infection, and children experiencing toothache have difficulty with eating, sleeping, and socialising. The consequences of dental decay can have wider impacts on society affecting children’s wellbeing, school readiness and attendance, as well as costs to the wider family, as parents/carers may have to take time off work to take children to dental and GP appointments.^2–4^

There is wide variation in the prevalence of dental decay in children across local authorities in England and national reports highlight the strong correlation with deprivation.^1^ Children living in disadvantaged communities having poorer oral health than those living in ones that are more affluent.^1,2^ This is evident in Plymouth, England, where local surveys carried out over a number of years have consistently shown Plymouth is a city with poor levels of oral health and significant oral health inequalities. In 2009, on an electoral ward basis there was more than a five-fold variation in dental disease levels in 5 year olds across the city, with over 55% of children in deprived wards affected by decay.^5^ The same pattern is observed in children (<16 years of age) having teeth removed under general anaesthesia, where there is more than a fourfold variation in the rates of extraction by ward.^3^ It has been estimated that children lost 3565 days of education in 2016/17 due to having teeth removed under general anaesthesia.^3^

Public Health England has provided recommendations and supporting evidence on interventions that improve oral health of young children and there is some evidence of effectiveness of targeted supervised tooth brushing, provision of toothbrush, and paste and community fluoride varnish schemes.^4^ However, there is less agreement on the most effective ways to deliver oral health education.^6^ Furthermore, oral health education does not feature prominently in the primary school curriculum in England despite the World Health Organisation’s Health Promoting School framework.^7^

It is recognised that achieving positive oral health outcomes for children is much broader than dental interventions alone and it requires the support and commitment of a range of partners. Integrating oral health education into school life and curriculum is an important objective and the purpose of this paper is to describe the development of a new oral health improvement programme in Plymouth, England, called “Open Wide and Step Inside”. The programme has been developed using a community engagement approach involving partners from the local authority, schools, health sector, and private and charitable organisations.

## Aims and methods

The “Open Wide and Step Inside” programme was developed in response to a clear need to provide effective oral health information, advice, and support to young children and their families in the city. The programme also supports teachers and schools to integrate the key oral health messages into Key Stage One of the National Curriculum. The programme comprises:

- A 15 min animation containing a 2 min brushing song and quiz
- A teachers resource pack
- A children’s classroom workbook
- A free toothbrush and toothpaste contained in a “Goody bag” with information on how to access local dental care, a brushing chart door hanger and 2 min timer
- “Open Wide and Step Inside” storybook
- Other resources such as classroom displays, bunting, and stickers

### Design and development of the programme

Each stage of the programme was designed collaboratively with partners using a community engagement approach, led by a consultant in dental public health and community officer following the principles of community engagement provided in NICE guidance.^8^ This has ensured the quality and content of the programme accurately reflects the needs of the communities it serves. Two focus groups with representatives from early years setting’s, primary schools, health visitors, school nurses, public health and dental professionals were used to define the project brief. Once a brief was agreed, schoolchildren were asked to provide ideas for a storyboard that could tell a story about “looking after” teeth. The winning entry formed the basis to the two main characters used in the film.

### The film

A creative content company (*The Moment*) was commissioned to develop a high quality contemporary animation working to a brief developed by the project leads. This was a highly creative process to develop the film characters, their attributes, and an accompanying script working with stakeholders to ensure the storyline was age/curriculum appropriate and aligned to evidence based oral health guidelines. The film tells the story of a Giant with toothache resulting from eating too many sweets, and the viewers discover he has never visited the dentist before. Geoffrey the Giant, with his mouse friend then embarks on an adventure to visit Daisy the fairy dentist. Along the way they meet other characters who each share a key oral health message. A quiz closes the animation testing what the children have learnt. The animation is fun, dynamic, and engaging in style and the characters form the basis of a “brand” that has been used to develop other assets within the programme.

### Brushing song

The film contains a two-minute brushing song that children can sing along to in the classroom. During the song the animation depicts teeth being coated with fluoride varnish, as well as promoting healthy food choices. The song was commissioned from a local music charity (*Plymouth Music Zone*) and children were involved in its development and production.

### Teacher and classroom resources

**Fig. 1:**
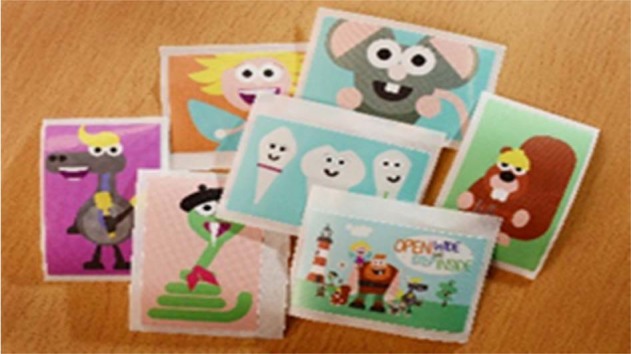
“Open Wide and Step Inside” resources

Classroom resources have been developed for Key Stage One of the National Curriculum integrating oral health more fully into Personal, Health and Social Education (PHSE). Developing resources such as lesson plans and activities was very much at the request of teachers who wanted more support to cover the key oral health messages they should be promoting in class (Fig. 1). The children are encouraged to take their individual workbooks home to share with their families. The resources have been refined over a number of iterations using focus groups with teachers for feedback and experts from the local authority education department.

### Implementation and participation

The programme has a targeted design working with schools in the most deprived wards of the city (based on the Index of Multiple Deprivation 2015). Schools were identified based on data from the local authority public health team and a target was set to engage with schools (*n* = 12) each year representing approximately 900 children in primary reception and year one classes (4–5 year olds). Established relationships already existed with some of the schools (due to other programmes such as supervised tooth brushing and fluoride varnish), whereas in others new relationships had to be developed and secured. The new programme was felt to be complimentary and supportive of existing activities. For the participating schools regular contact is maintained by the delivery team to ensure efficient implementation of the programme.

**Fig. 2:**
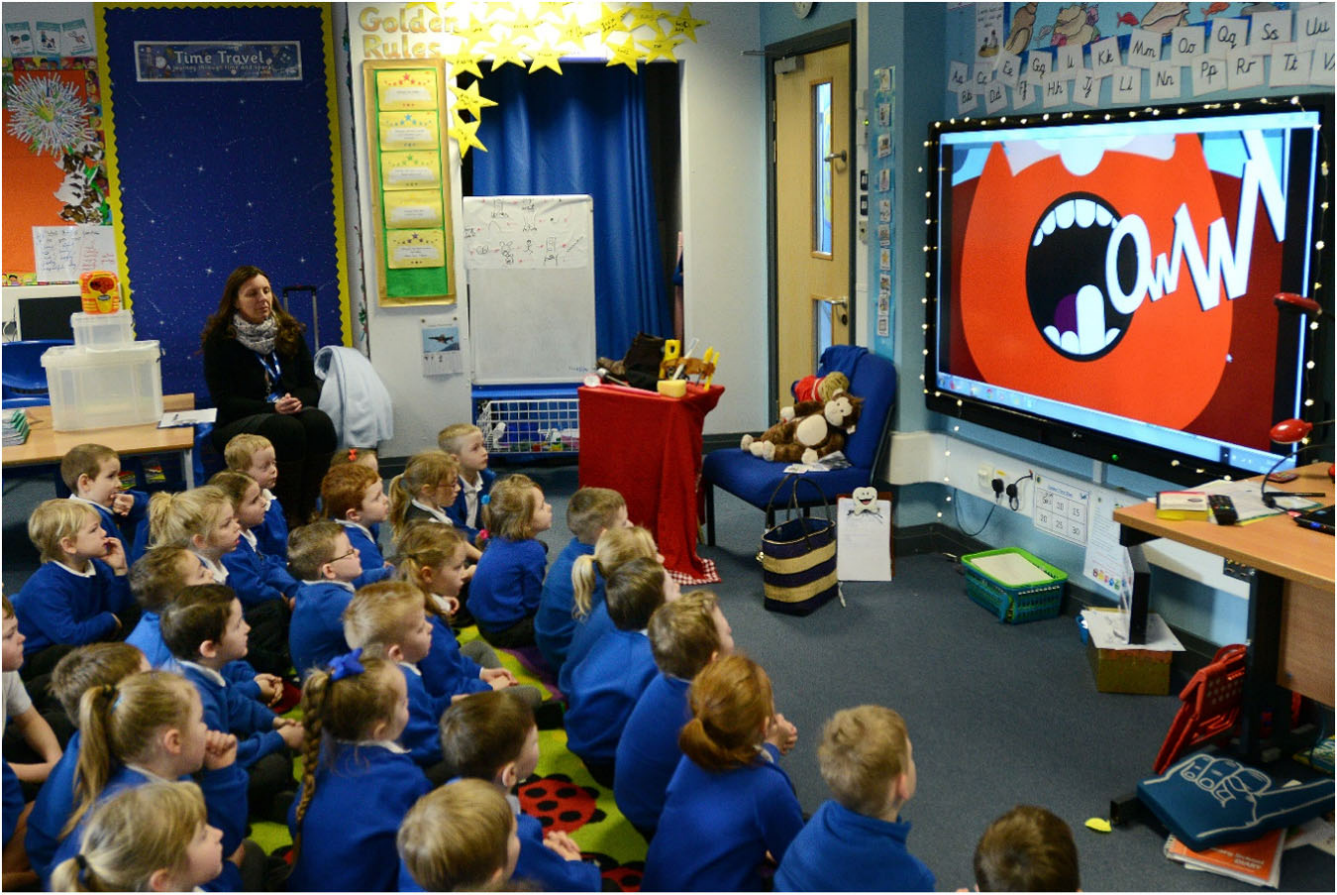
Open Wide and Step Inside animation being shown in the classroom (permission to use photograph)

“Open Wide and Step Inside” is delivered in two phases. The first is to gain agreement with the schools to deliver the programme in the classroom (Fig. 2) and to introduce the resources to the teaching staff as part of their engagement in the programme. The classroom session is led by an Oral Health Education qualified dental nurse, and it is theatrical in approach, making use of props related to the animation storyboard to introduce the characters to the children and to reinforce the key oral health messages. The brushing song and quiz in the animation encourage children to actively take part. Simple baseline data is collected on the children’s existing knowledge by asking five questions and responses are collected using “*turning point*”® technology. Each child receives a “Goody bag” with a toothbrush and toothpaste and other items. Phase two takes place 3–4 months later when a follow-up visit takes place. The children are asked the same five questions again to test what knowledge they have retained, and this is followed up with an interactive classroom session about healthy food choices and being sugar “smart”. At the end of the follow-up visit the children are given an “Open Wide and Step Inside” storybook to take home.

### Process evaluation

An evaluation framework was developed to understand how well the project performed across the following areas:

- Delivery—was the programme implemented as planned and communicated to partners?
  1. Programme reach—did the intervention reach the target group?
  2. Programme acceptability—were children, parents, and teachers satisfied with the intervention?
  3. Programme integrity—were all aspects of the intervention delivered as planned?
- Effectiveness—were the intended objectives and outcomes achieved?
- Quality—were the programme resources of good quality?
- Efficiency—were resources being used?

**Fig. 3:**
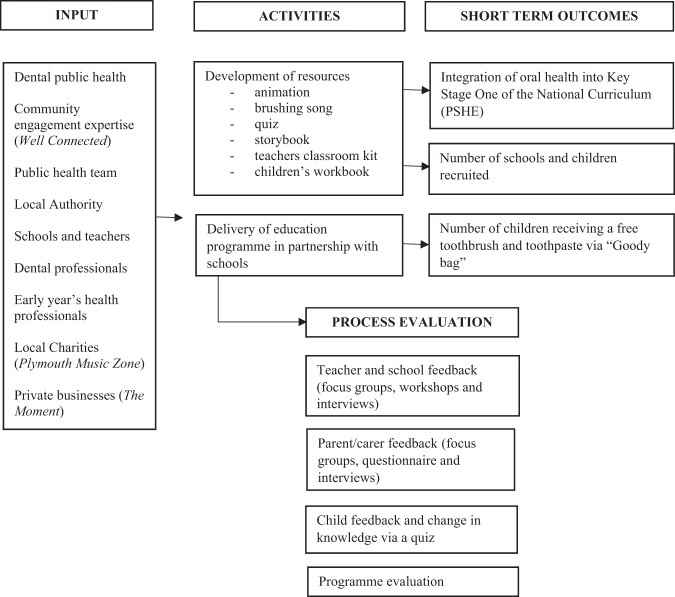
Logic model for programme. Please see separate attachment

Due to the multiple interventions involved in “Open Wide and Step Inside”, a logic model was developed to identify inputs, activities, and process and outcome measures (Fig. 3). The evaluation is an ongoing process and the framework continues to evolve with a different focus each year as the programme continues. An independent investigator carried out the initial process evaluation in the first year of the programme (2014/15). Not all feedback sought from questionnaires and in focus groups with stakeholders (parents, children, and teachers) is presented in the paper. All data collected as part of the evaluation are anonymised.

## Results

### Delivery of the programme

**Fig. 4:**
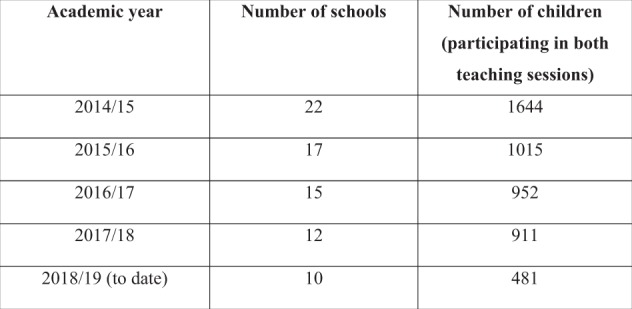
Participation rates

The initial objective was to engage with 12 primary schools (*n* = 12) in Plymouth each year targeting ~900 4–5-year-old children. In the first year of the programme (2014/15) at least 12 schools were successfully recruited (Fig. 4) and in subsequent years the number of schools has met or exceeded the target number.

As the intervention is education focused and integrated into curriculum, no formal consent is sought, and, therefore, participation rates among the children are high (in excess of 98%) meaning the programmes reach is acceptable and targets the children considered at high risk. Programme acceptability and integrity was also achieved in the first year. Feedback from schools was very positive on the communication, planning and organisation of the programme.

“Staff are so grateful for efficient organisation. The communication and organisation was great and the children loved it!” (Primary School Head Teacher)

In the early phases of the programme parents were also invited to view the film and attend the session with their children, and there was some uptake of this option.

### Effectiveness

#### Impacts on children

There is overall agreement that children enjoy the programme based on comments collected in the school as part of the classroom exercises, and the fun and interactive style of the film is considered engaging and informative. There have been no negative issues reported by any child, parent, or school.

“The children loved feeling special and enjoyed the theatrical experience” (Primary School Teacher)

“Children who sometimes find it hard to focus without talking were transfixed” (Primary School Teacher)

**Fig. 5:**
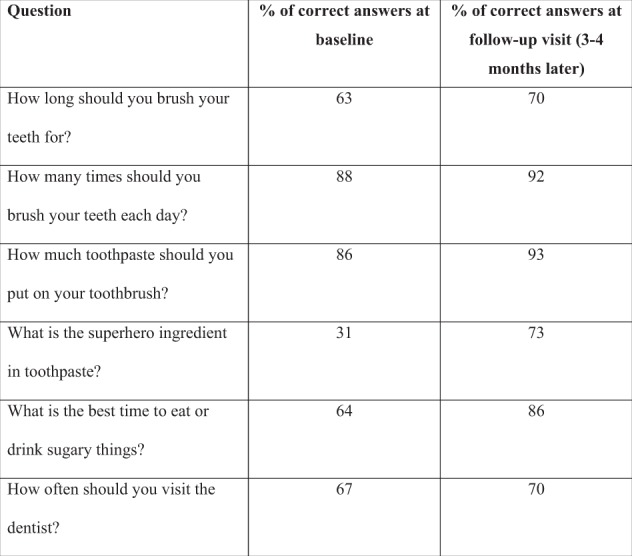
Response rates from children (*n* = 911) for the most recent academic year 2017/18

The data collected each year (from academic year 2015/16 onwards) show the majority of children (>80%) are able to recall the correct answers to the five key questions contained within the quiz 3–4 months later at a follow-up visit (Fig. 5). Children are generally knowledgeable about tooth brushing norms whereas answers relating to the importance of fluoride, and when is the best time to eat or drink sugary things score lower.

#### Impacts on parents

Impacts on parents has been more difficult to measure although some attempts were made in the early phases of the programme to engage with families (*n* = 127 parents) through feedback sessions in the schools using an independent evaluator. Feedback was based on three simple questions (1) did your child enjoy the film, (2) did your child show you the “Goody bag”, and (3) have you made any changes to how you look after your child’s teeth? Feedback was positive across all three questions with most parents noticing a change in their children’s behaviour. A number of parents commented:

“She said she liked it because of the tooth brush song” (Parent)

“He loved his “Goody bag” and so far he is using his timer and is enjoying marking his chart when he has done his teeth” (Parent)

“This film and the contents of the bag have motivated my child to brush her teeth better and twice a day. We had been having difficulty previously as she was very resistant to brushing. It has made a BIG difference, particularly the timer” (Parent)

Attempts to make data collection a more routine part of the evaluation have been hampered by project budget and resource, and the disruption it creates to the school day. Parents are still encouraged to provide feedback via the school and we are currently investigated ways to collect this in the future via a reply slip in the “Goody bag” or online.

#### Impacts on schools and teachers

Schools have reported the positive impact the programme has had on school culture and curriculum. For many it has resulted in wider benefits such as providing additional evidence for Healthy School status, informing Ofsted reports and involving families that are sometimes hard to engage with. The initial driver for the programme was a request from teachers for more support in delivering key oral health prevention messages as part of the curriculum and it appears that schools are satisfied with the resulting products.

“Children and staff were completely buzzing about it yesterday. Literally my whole class say they brushed their teeth last night and this morning, that’s got to be a first” (Primary School Teacher).

### Quality

The quality of resources has been a key driver in developing the programme and these were designed in collaboration with stakeholders from different sectors (public, private, third, and charitable sectors) using an iterative process involving focus groups and workshops. Feedback from schools and teachers has been positive with high levels of satisfaction regarding the quality level of materials and the delivery of the programme overall. One piece of constructive feedback received from teachers during piloting of the classroom resources was that they felt they were pitched too high for Key Stage One. In response, they were revised accordingly seeking expert education advice from the local authority to achieve conformance to national curriculum guidelines.

### Efficiency

Throughout the evaluation period, stakeholders have considered the programme efficient in its delivery with minimal impact on schools or variation reported in delivery between schools. Schools particularly commented in feedback on the benefits of using the same OHE dental nurse to maintain consistency, relationships with the children, and the quality in educational delivery. Changes continue to be made to refine processes and make improvements where feasible. A number of changes are planned to make the programme more sustainable in the future including developing a commercial model for wider dissemination, creation of a website, translating material into different languages, and increasing brand awareness through social media use.

## Discussion

The “Open Wide and Step Inside” programme was developed to complement and enhance existing oral health improvement activities (targeted supervised tooth brushing and fluoride varnish schemes in the schools) by adding another dimension to the interventions already available. The primary driver was to develop sustainable resources that could be used to engage children in an exciting and fun way about oral health, and to support teachers by integrating oral health into the national curriculum at Key Stage One. While existing resources with a similar aim already exist, the authors felt development of a programme and “local” brand (for example, the film contains local landmarks) through co-design and a community engagement approach would result in higher participation and greater success in implementation. The programme also addresses some of the gaps that exist in current services in the city.

The main outcome is the real enthusiasm and excitement demonstrated by the children and schools participating in the programme evidenced through various feedback routes and the high participation rates. In addition, the majority of children (>80%) show improvements in knowledge across the five key questions between baseline and follow-up 3–4 months later. Participation rates are achieved by making the programme part of the fabric of school life and while no formal consent process was put in place due to its educational approach, parents were given the option to opt their child out but none have to date. There are challenges to working in schools, and these have been reported in other programmes which can limit success and sustainability due to time, space, and organization requirements.^9,10^ We recognise some of these challenges and support the view that there is often an underestimation of the time (and resource) needed to engage and maintain a positive relationship with schools. Our community engagement “first” approach is designed to overcome these challenges by involving schools at the very start of the project cycle. An element the authors feel is crucial to relationship building and success is trust. Once trust was established with many of the schools and they could see it would enhance their curriculum with minimal operational impact on the busy school day, they have been enthusiastic participants. There have also been wider benefits to the schools involved with many using participation in the programme to support their own Ofsted inspections, health and wellbeing strategies, and healthy schools status.

One of the most significant challenges has been evaluating the impact of the programme due to its multiple components and the young age of the children involved. In the first year of the programme (2014/15) data were collected from children in a questionnaire via the classroom although this proved administratively challenging and resource intensive for all parties. Due to the age of the children the reliability of the results was also questionable. The new system of using an interactive quiz and individual response clickers that each child uses has improved the efficiency and accuracy in evaluation however, it is has to be acknowledged that results may still have a degree of unreliability due to the young age of the children involved.

There has been consistent calls to develop new and innovative ways of delivering oral health education that engage children in the key oral health messages from an early age as possible.^4,6^ It is recognised that traditional oral health education alone is ineffective in improving oral health unless exposure to fluoride is included.^6,11^ For this reason, each child participating in the programme receives a free toothpaste and toothbrush supporting Public Health England’s advice on distribution of these items to high-risk groups^11^ along with other supporting materials to use at home. These include a reading storybook based on the animation, which is provided to children at the follow-up visit, to reinforce the key oral health messages at every opportunity in the home, accepting that children at this age are under direct parental control. Additionally in the majority of schools other schemes operate including supervised tooth brushing and fluoride varnish application creating a suite of complementary interventions addressed at different intervention levels. This multi-component approach is recognised as being more successful in health promotion than standalone initiatives.^8,12^

The “Open Wide and Step Inside” programme is creative and engaging in style and approach, and it has created awareness about oral health in the city. The local authority has now incorporated poor oral health into its Child Poverty Action Plan recognising the importance of the issue and the “Open Wide and Step Inside” programme is central to its delivery.

There are some limitations to the service evaluation, as it has focused on process evaluation and short term outcomes such as change in knowledge and not on longer-term oral health outcomes and behaviour change. In addition, there may be the potential for bias in the results, as parent feedback may only represent more motivated and engaged families. Furthermore, the evaluation framework is pragmatic in approach to account for ongoing programme adjustments to improve its delivery, meaning comparisons between years cannot be made.

## Conclusions

Developing the “Open Wide and Step Inside” in partnership with local stakeholders has resulted in a programme with high acceptability and integration into the local health and education system. One of the strengths of the programme is its adoption of NICE guidance with respect to community engagement, and its co-design with participants, resulting in a programme aligned to the local needs of communities. It complements existing oral health activities in the city and while there are limitations to this evaluation, feedback received indicates the programme is working successfully and achieving its objectives of improving oral health integration into curriculum in local schools in a fun and engaging way.
